# Hysteretic Behavior of Geopolymer Concrete with Active Confinement Subjected to Monotonic and Cyclic Axial Compression: An Experimental Study

**DOI:** 10.3390/ma13183997

**Published:** 2020-09-09

**Authors:** Huailiang Wang, Yuhui Wu, Min Wei, Lang Wang, Baoquan Cheng

**Affiliations:** 1 College of Civil Engineering and Architecture, Guangxi University, Nanning 530004, China; whuailiang@gxu.edu.cn (H.W.); gxuwyh@st.gxu.edu.cn (Y.W.); gxuwm@st.gxu.edu.cn (M.W.); wanglang@st.gxu.edu.cn (L.W.); 2 Key Laboratory of Disaster Prevention and Structural Safety of Ministry of Education, Nanning 530004, China; 3 School of Civil Engineering, Central South University, Changsha 410075, China; 4 Department of Civil and Environmental Engineering, The Hong Kong Polytechnic University, Kowloon, Hong Kong 999077, China

**Keywords:** geopolymer concrete, triaxial compression, hysteretic behavior, active confinement, monotonic and cyclic loading, experimental study

## Abstract

This paper investigated the performance of actively confined geopolymer concrete (GPC) through experiments. The mechanical properties of GPC under triaxial stress states were analyzed and discussed from the prospects of failure modes, axial peak stress and strain, monotonic and cyclic constitutive relationships. The experimental results demonstrated that the loading modes (monotonic loading and cyclic loading) had little effect on the failure mode and axial peak stress and strain. The improvement of the strength and ductility of GPC with the increase in confinement level was consistent with that of the conventional cement concrete while the strain enhancement of confined GPC was lower than that of confined conventional cement concrete at the same confinement level. The curves of the monotonic stress–strain and the envelop of cyclic compression were predicted through Mander’s model with good accuracy. The unloading/reloading models proposed by Lokuge were modified and the predicted cyclic hysteresis curves for actively confined GPC were in good agreement with the cyclic compression results. Findings from this study provide references for the application of geopolymer concrete.

## 1. Introduction

It is widely accepted that concrete is the main building material for infrastructure construction and development, it is generally the production of ordinary Portland cement (OPC) and other ingredients. Cement production consumes a lot of natural resources and releases a lot of greenhouse gas to the environment [1,2,3]. As reported in [4], the manufacturing process of OPC contributes to 5–8% of global greenhouse gas emissions. Geopolymer concrete (GPC) is also known as “Alkali-activated concrete (AAC)”. Its cementitious materials are generally generated by activating waste industrial and agricultural byproducts by strong alkalis, such as granulated blast furnace slag powder (GBFS), fly ash (FA), rice husk ash (RHA), and metakaolin. It has attracted much attention as an environment-friendly alternative cementitious material to OPC concrete. The comparable mechanical properties and durability of GPC have been widely reported [5,6,7,8,9]. Noushini et al. [10] found that the stress–strain behavior of low-calcium FA-based GPC under uniaxial compression loading was similar to that of heat-cured OPC, and reported a more brittle post-peak response as well as higher curing temperature results for FA-based GPC. However, the higher curing temperature results in a more ductile post-peak behavior of heat-cured OPC. According to the investigation, the compressive strength of FA-based GPC ranged from 27.4 to 62.3 MPa, the strength of heat-cured OPC ranged between 42.6 and 64.5 MPa at age 28 days. Due to the demanding heat-curing conditions, the in-situ field application of FA-based GPC structural elements was limited. Xie et al. [11] found the combination of GBFS and FA can realize the room-curing GPC and provide excellent synergetic effects to the mechanical performance for the GPC despite the use of recycled coarse aggregate. In their investigation, the compressive strength of FA-GBFS-based GPC ranged from 25.2 to 79.6 MPa, and compressive strength increased with the increase in GBFS content in the binder material. However, with the increase in GBFS, the descending part of the stress–strain of GPC became steeper. Thomas et al. [7] also found that the stress–strain behavior of GPC (strength ranging from 29.5 to 52.6 MPa) with GBFS as the solo binder showed a rapid decline during the post-peak part. The microstructure analysis in [12] showed that the calcium content available for geopolymerization increased as the amount of GBFS increased. The polymerization of blended fly ash and GBFS with alkali activator not only produced Sodium Alumino Silicate Hydrate gel (N-A-S-H networks) but also produced Calcium Alumino Silicate Hydrate gel (C-A-S-H networks). Therefore, it can be concluded that the blended FA-GBFS as a binder to produce GPC had comparable compressive strength at ambient temperature and open the scope of the use of GPC as the structural members’ in-situ field. A great number of reinforced GPC structural applications such as beams, columns, slabs, and multi-story frame structural elements have been reported [13,14]. In the literature, the authors also listed various construction projects using both precast and casting in-situ of GPC such as the precast retaining wall, precast bridge decks and boat ramp, steel-reinforced foundation slabs, and footpaths using cast in situ GPC.

As mentioned above, the application of GPC in practical engineering is very appealing. Nonlinear analysis and design of GPC structures require a reliable and accurate material model of GPC, which can only be obtained by conducting multi-axial experiments on structural GPC. On the other hand, due to the inherent brittleness of GPC and the high demand for ductility of GPC structures subjected to extreme earthquake action, structural members such as GPC-filled steel tube and fibre reinforced polymer (FRP) tube, and spiral columns made of GPC are recommended. The core concrete in these structures is also subjected to a multi-axial stress state and even the hysteretic loading scenario. The strength criterion and constitutive model of concrete material under multi-axial stress conditions can be used to establish a reasonable finite element model for structural analyzing. Therefore, it is necessary to establish a complete constitutive model including both monotonic and hysteretic behavior of laterally confined GPC. At present, there are relatively few studies on the triaxial or cyclic mechanical performance of GPC, especially the actively confined GPC [15]. Ozbakkaloglu et al. [16] studied the behaviors of steel tube and FRP-confined FA-based GPC. It was found that the strength and strain enhancement ratios of confined GPC were slightly lower than those of conventional cement concrete at a similar confinement level. GPC exhibits less deformation and ductility in the axial direction than conventional concrete under the same confinement. Gholampour et al. [17] studied the monotonic stress–strain behavior of GPC under active confinement (provided by hydraulic servo testing machine). They also found that the stress–strain curve of GPC under a low confining level was quite different from that of the cement concrete, and the strength and deformation properties of GPC under active confinement were related to the material composition and curing condition. The stress–strain curve of the GPC becomes steeper because of the addition of GBFS after heat curing. In the literature [18,19], authors conducted the cyclic compressive test of confined cement concrete, and developed several hysteretic stress–strain models of confined cement concrete which were verified with the test data of loading/unloading/reloading paths.

However, the constitutive behavior of actively confined GPC under cyclic loading has not been fully investigated yet. To fill the research gap, this study aims to investigate the constitutive behavior of confined GPC subjected to monotonic and cyclic compression. Based on experimental results, new mathematical models of describing the monotonic and cyclic constitutive behavior of GPC with lateral active confinement were developed, respectively. The findings deepen the understanding of the mechanical properties of GPC, and facilitates the application of these sustainable building materials.

## 2. Materials and Methods

### 2.1. Materials and Specimen Preparation

The binding materials used in this study included FA (Guodian Nanning Liujing thermal power plant, Nanning, China) and GBFS (Guangxi Liuzhou Iron and Steel Group, Liuzhou, China). The chemical compositions of the FA and GBFS used to make GPC were listed in Table 1. Alkali activator was a mixture of sodium hydroxide solution (SH) and sodium silicate solution (SS) with a solute ratio of 1:2.5. The concentration of sodium hydroxide solution was 6 Mol/L, the relative density and volume concentration of sodium silicate solution were 1.35 and 38%, respectively. Natural river sand with a fineness modulus of 2.4, the apparent density of 2630 kg/m^3^, and water absorption rate of 0.8% was used as fine aggregates for all concrete mixtures. The crushed limestone, with the particle diameter ranging from 5 to 20 mm and the apparent density of 2684 kg/m^3^ were selected as coarse aggregates for GPC. The designed strength grade of the concrete mix was 30 MPa, which was measured by 150 × 150 × 150 mm standard cubic specimen according to the Chinese standard GB/T50081-2002 [20].

The mixture proportions used in this study are listed in Table 2. The water in the mixture included the water used in the preparation of the alkali activator solution and the additional water added to the mixture during the mixing process, so the water-binder ratio was 0.39 in this study. The GPC mixing procedure began with mixing the coarse, fine aggregates with the blended powder raw material for approximately 3 min. Then, the alkaline activator solution was added and mixed for an additional 1 min. Finally, the superplasticizer (1.4 wt.% of the binding materials) was added with the additional water, mixing concrete for 2 min again until the mixture was homogenized. All specimens were put into steel molds and vibrated on the shaking table for about 10 s. Three 150 × 150 × 150 mm cubes were cast to test the strength grade, and forty-eight 100 × 200 mm cylinders were cast for the uniaxial and triaxial tests. All samples were demolded after 24 h, then cured in the curing room with the temperature of 25 ± 5 degrees centigrade and relative humidity of 50–55% for 28 days. After 28 days of curing at ambient temperature, the standard cube compressive strength tests (150 mm cube side) were carried out for the GPC made above. The test results showed that the average cubic compressive strength of GPC in this study was equal to 30 ± 3 MPa.

### 2.2. Experiment Design

**Figure 1:**
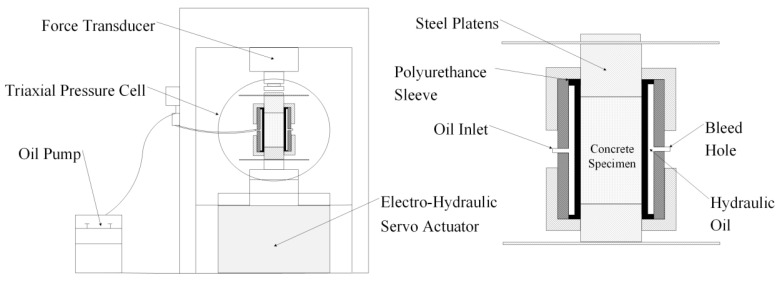
Schematic diagram of the triaxial compressive cell.

**Figure 2:**
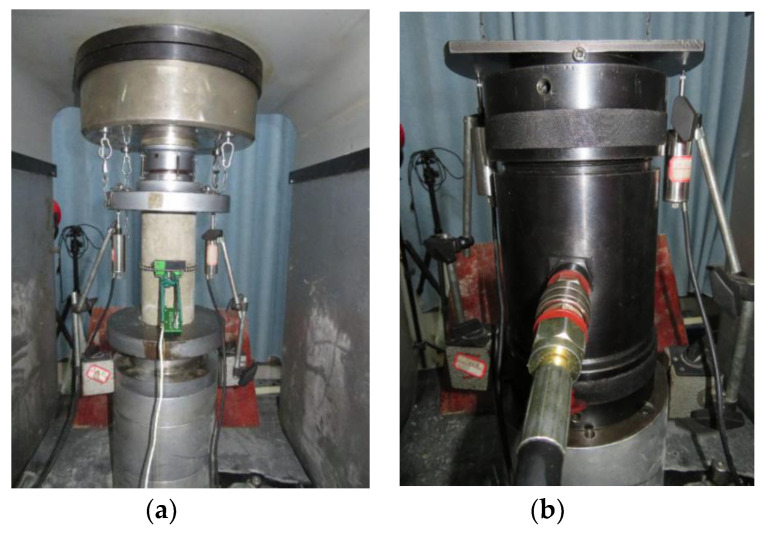
Test setup: (**a**) uniaxial compression; (**b**) triaxial compression.

**Figure 3:**
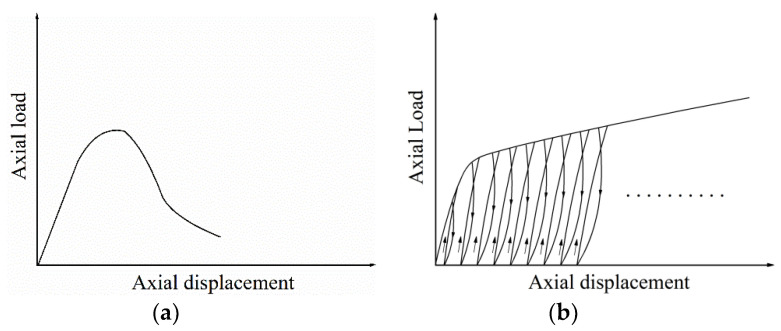
Loading scheme: (**a**) monotonic load; (**b**) cyclic load.

Uniaxial and triaxial compression tests under monotonic and cyclic loading were carried out on the RMT-201 rock-concrete material test system developed by the Wuhan Institute of Rock and Soil Mechanics, Chinese Academy of Sciences, and SIMENS company (Munich, Germany). The schematic diagram of the device is shown in Figure 1. The main component of the device was a Hoek pressure chamber which can accommodate a cylindrical sample with the size of 100 mm × 200 mm. During the test, the oil was pumped into the Hoek cell through the vent valve to provide uniform circumferential pressure for the sample. The axial compression load was applied to the sample through the steel spherical hinges at the upper and lower ends of the pressure chamber. For the uniaxial compression test, two linear variable differential transformers (LVDTs) and a circumferential displacement gauge were installed on surfaces of the cylindrical sample at the axis parallel position and the middle height position, respectively, as shown in Figure 2a, to measure the axial and circumferential displacement. The uniaxial compression test was carried out in displacement controlling mode with the rate of 0.01 mm/s until the sample failed. For the triaxial compression tests, as shown in (Figure 2b), two external LVDTs and one circumferential displacement gauge were still used to measure the axial and circumferential deformation. Before the concrete sample was inserted into the rubber sleeve of the Hoek cell, a thin layer of lubricating wax was applied on the surface of the circumferential displacement gauge and the steel conducting wire to reduce the friction between the gauge and the rubber sleeve [21,22,23,24,25]. In this study, two load schemes were used and the lateral pressure remained constant during the triaxial compression test at designed values of 6 MPa, 12 MPa, 18 MPa, or 24 MPa. Load path 1 was the monotonic load which included applying the lateral pressure at a loading rate of 0.1 MPa/s and applying the axial load at a displacement rate of 0.01 mm/s until the specimen failed (Figure 3a). Load path 2 was the cyclic load, including several complete unloading/reloading cycles at the specified displacement (Figure 3b). It should be noted that to prevent any unnecessary movement of the specimen during unloading, this process should be stopped when the load value is in equilibrium with the confining pressure.

## 3. Results and Discussions

### 3.1. Failure Patterns of Unconfined and Confined GPC

**Figure 4:**
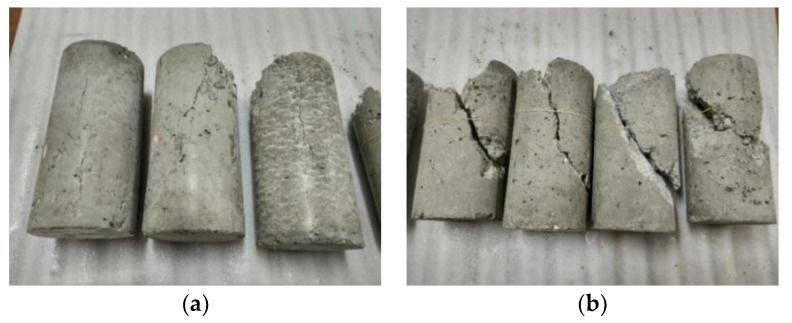
Failure modes: (**a**) uniaxial compression; (**b**) triaxial compression (from left to right *σ*_1_ = 6, 12, 18, 24 MPa).

Figure 4 demonstrated the typical failure patterns of unconfined and confined GPC samples. It can be seen that in the case of unconfined uniaxial compression, samples mostly presented vertical microcracks; under lateral confining stresses, samples mostly presented the shear failure mode with a major diagonal shear crack in the middle of the specimen. Under higher lateral confining stresses, the width of the oblique crack increased significantly at axial peak stress, and the expansion phenomenon occurred in the middle of the specimen. The loading form (monotonic or cyclic loading) had no obvious influence on the failure mode of GPCs. These failure patterns were also consistent with those observed in cement concrete in conventional triaxial compressive tests [26,27]. The uniaxial compressive strength of high strength cement concrete [26] was 68 MPa and the uniaxial compressive strength of recycled aggregate cement concrete [27] was 19 MPa. The specimens used in the literature above were cylinders with the size of 100 × 200 mm, whose sizes were same as in this study. By comparing with these failure modes of cement concrete, it can be found the failure modes of OPC and GPC after triaxial compression were only bound to laterally confined level (*σ*_l_/*f*_c_) and not relevant to the uniaxial compressive strength (*f*_c_) of concrete.

### 3.2. The Peak Value of Axial Stress and Strain

Table 3 shows the measured peak value of axial stress and strain of GPC under different lateral confinements. It can be seen that the peak stress ratio (*f*_cc_/*f*_c_) and peak strain ratio (*ε*_cc_/*ε*_co_) increased with the increase in the circumferential confinement *σ*_l_/*f*_c_, which was similar to the test result of conventional cement concrete. When the circumferential confined stress increased from 6 to 24 MPa, the peak compressive stress and strain increased by 96% and 205.7% to 330.4% and 1055.6%, respectively, compared to the uniaxial strength and peak strain under uniaxial compression. The axial load forms (monotonic and cyclic loading) had no obvious influence on the strength and deformation enhancement.

Several models [17,28,29,30,31,32] to predict the compressive peak strength and corresponding axial strain were listed in Table 4.

**Figure 5:**
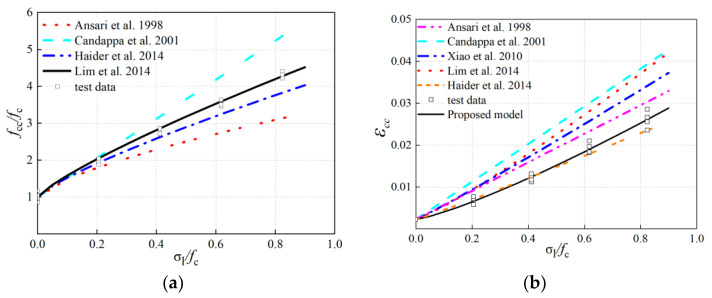
Comparison of experimental results of peak strength and strain with predicted values of different models: (**a**) peak axial stress(*f*_cc_); (**b**) peak axial stress(*ε*_cc_).

It is worth noting that the above models were proposed for different types of concrete. Figure 5a showed a comparison between the above strength prediction models and the experimental values of this study. It can be found that the prediction model [31] was very close to the test results in this study. Aliakbar et al. [17] also verified that the strength prediction model in Lim et al. [31] was applicable to confine both ambient- and oven-cured GPC; the strength prediction model [31] was adopted in this study.

Figure 5b indicated that the predicted trends of the considered axial peak strain prediction models were very similar, that was, the peak axial strain increased with the increase in confining pressure. However, most axial peak strain prediction models for cement concrete gave too high predicted values, compared with the experimental values of this study. The model in [27] was close to the experimental results of this study at low confinement levels, but not very accurate and reliable in the case of high confinement levels. Therefore, the following prediction model was proposed in this study:

(1) $$\varepsilon_{cc}=\varepsilon_{0}[1+13.446{\left(\frac{\sigma_{l}}{f_{c}}\right)}^{1.2299}]\;\;where\;\varepsilon_{0}=0.00225$$

It is noted that the formulation above was figured out by experimental data with the least-squares method. Figure 5 demonstrated that the peak strength model of actively confined GPC was similar to the conventional cement concrete. However, the peak axial strain of confined GPC was smaller than that of conventional cement concrete at the same confinement level. This can be explained by the observation that the interface transition zone of cement concrete is a weak link: under uniaxial compression, microcracks propagate rapidly from here. Moreover, after confining pressure was applied, the crack propagating speed decreased and the strength of triaxial compression increased, based on the literature [12], thermal curing FA-based GPC showed few interfacial transition zones, less initial microcracks and defects, and the improvement of strength and deformation under triaxial compression was smaller compared to that of cement concrete. In this paper, the GBS-FA-based GPC was cast and cured at room temperature. Although the three-dimensional network microstructure of C-A-S-H was formed, the GBFS particles had higher microcracks that lead the performance of GBS-FA-based GPC to be similar to cement concrete. Therefore, the strength model of cement concrete, especially the multi-axial strength model of high strength concrete, is also suitable for GBS-FA-based GPC, and the lower peak strain is foreseeable because of more microcracks internally.

### 3.3. Monotonic Constitutive Behavior

**Figure 6:**
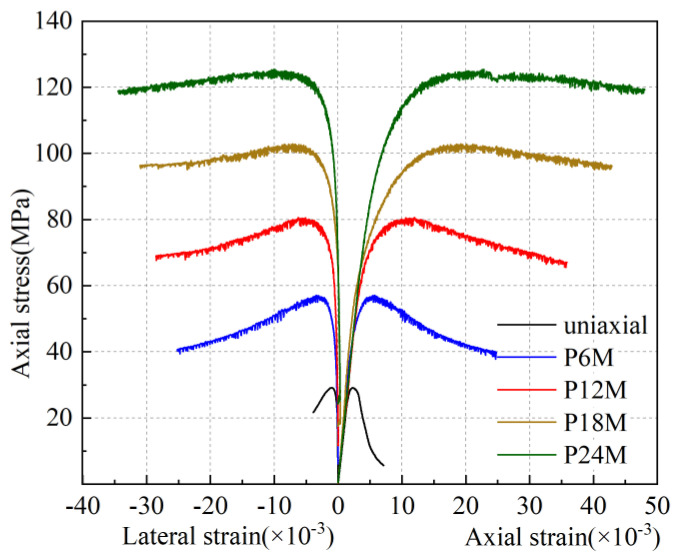
Constitutive relation of confined GPC subjected to monotonic compression.

Figure 6 illustrated the typical monotonic stress–strain responses of the GPC subjected to uniaxial compression and triaxial compression with various levels of confining lateral stress. Each group of curves was the average value of two measured identical specimens, including lateral additional strain–axial stress curve and axial additional strain–axial stress curve. As can be seen under the uniaxial compression and the confinement level of *σ*_l_/*f*_c_ = 0.2, the stress–strain curves had obvious ascending and descending branches. With the increase in the confinement level, the curve gradually became flat, and the post-peak descending part became shallower, which is similar to the test results of cement concrete [33] and GPC [32], when the confinement ratio *σ*_l_/*f*_c_ was greater than 0.4, the GPC in this study changed from brittle to ductile behavior. It is worth noting that only one group of the higher confining levels of GPC was conducted by Haider (2014) which was greater than 0.4, while the test of this study had two groups: the confinement ratios were 0.6 and 0.8.

**Figure 7:**
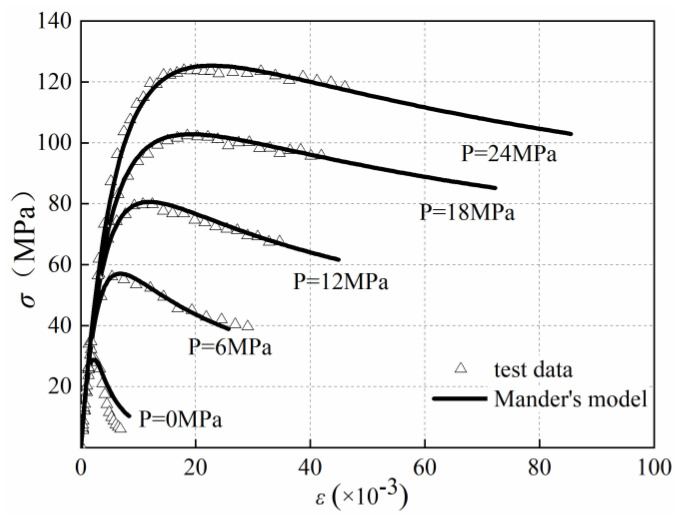
Monotonic axial stress-strain relation predictions of confined GPC.

Many stress–strain models had been put forward for the cement concrete under lateral confinement [31,33,34]. These models were very useful in the finite element analysis of the structural members confined by elastic materials, such as FRP fabric, spiral hooping, infilled steel tubular columns, anchorage area of prestressed concrete structure, beam-column joints, etc. In this study, a modified model proposed by Mander et al. [34] was adopted, because Mander’s model was simple in form and was the most widely used, it can also be used for the envelope curve simulation under subsequent cyclic loading conditions. The modification was based on experimental results, which can reach the best potential of empirical and semi-empirical formulations. The modified model in this study was as follows:

(2) $$\frac{\sigma_{c}}{f_{cc}}=\frac{r\times \frac{\varepsilon_{c}}{\varepsilon_{cc}}}{r-1+\left(\frac{\varepsilon_{c}}{\varepsilon_{p}}\right)r}$$

(3) $$r=\frac{k_{r}\times E_{c}}{E_{c}-E_{sec}}=\frac{k_{r}\times E_{c}}{E_{c}-f_{cc}/\varepsilon_{cc}}$$

(4) $$E_{sec}=f_{cc}/\varepsilon_{cc}$$

(5) $$k_{r}=\sqrt{f_{c}/30}$$

where *k*_r_ was a parameter related to the concrete strength, which was introduced in [35] to modify the descending section of the curve, and *E*_c_ was the elastic modulus of concrete. Figure 7 showed a comparison between the Mander’s model and monotonic loading test results. The model and test results were basically in good agreement except the descending curves in the case of low confinement.

### 3.4. Stress–Strain Behavior under Cyclic Loading

**Figure 8:**
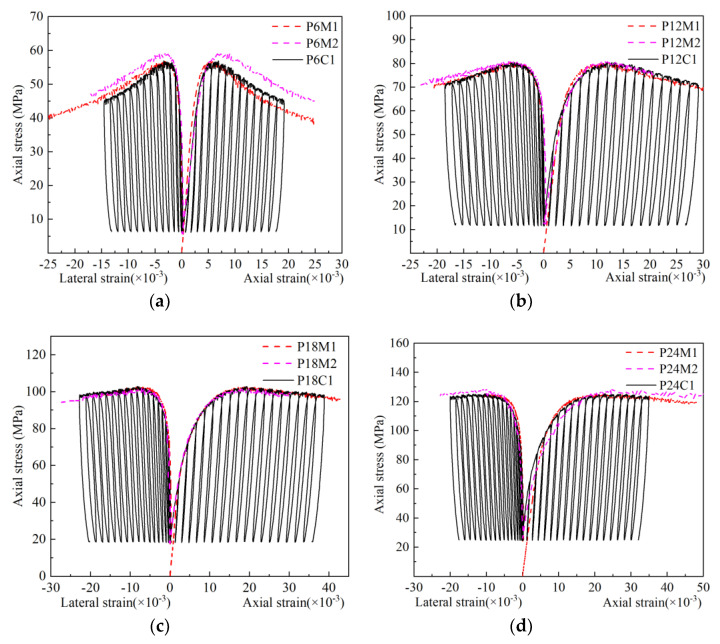
Cyclic axial and circumferential stress–strain curves of confined GPC: (**a**) *σ*_l_/fc = 0.2; (**b**) *σ*_l_/*f*_c_ = 0.4; (**c**) *σ*_l_/*f*_c_ = 0.6; (**d**) *σ*_l_/*f*_c_ = 0.8.

Figure 8 indicated the typical stress–strain behaviors of the confined GPC subjected to cyclic compression. In Figure 8, the axial compressive stress and strain were considered to be positive as the monotonic loading curves in Figure 7. For the sake of comparison, two sets of monotonic stress–strain curves of identical specimens from the same condition of confining pressure were placed in the same Figure. It can be seen from Figure 8 that the line connecting the peak points of each complete cyclic curve, which was the envelope line, was almost coincided with the monotonic compressive stress–strain curve. This conclusion had been confirmed by the uniaxial cyclic compression test [36], and the test results of actively confined cement concrete including steel tube infilled concrete [26,37], and passively confined concrete including various FRP confined concrete [19,38]. The experimental results showed this conclusion can also be applied to the actively confined GPC.

**Figure 9:**
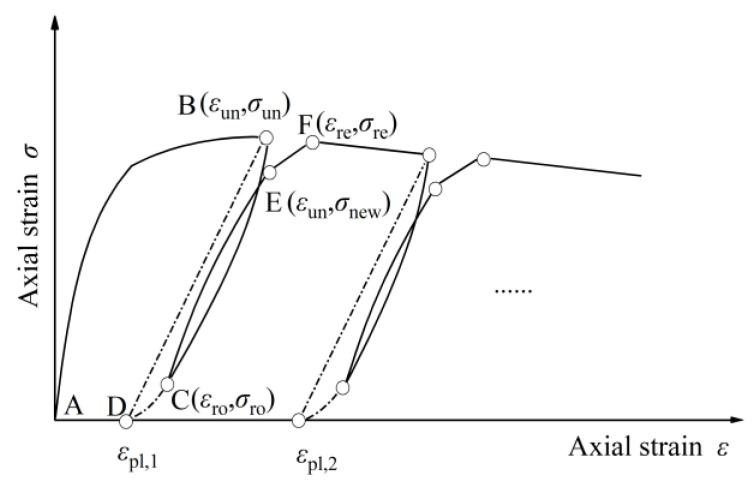
An analytical constitutive model for the cyclic behavior of concrete.

According to the test result, the general schematic view of the axial stress–strain constitutive relation of actively confined GPC under cyclic compression including the unloading and reloading parts was shown in Figure 9. The unloading component was from the unloading beginning point (*ε*_un_, *σ*_un_) on the envelope curve to its endpoint (*ε*_r0_, *σ*_r0_), which was estimated by the reverse stress–strain model of compressive loading; the reloading components were composed of the initial nonlinear curve and the linear transition curve until the point (*ε*_re_, *σ*_re_) on the envelope curve. The coordinates at the junction of the two reloading lines were defined as (*ε*_un_, *σ*_new_). The possible intersection point between the unloading curve and the *x*-axis was also the key point of the cyclic loading hysteretic constitutive model, where the strain corresponding to the zero stress point was defined as the residual plastic strain, *ε*_pl_.

In previous studies, different unloading/reloading models [18,19,34,35,36,37,39] had been provided to predict the cyclic constitutive behavior of confined conventional cement concrete. In the following part, the Mander’s [34] and Lokuge’s [18] hysteretic constitutive model will be modified to obtain the predicted cyclic compressive stress–strain curve of confined GPC, and compare it with the cyclic stress–strain curve obtained from the experiment. The residual plastic strains, common and reloading points were expressed by Equations (7)–(13), as follows:

(6) $$\varepsilon_{pl}=\varepsilon_{un}-\frac{\sigma_{un}}{E_{c}}\;\;where\;0\leq \varepsilon_{un}\leq \varepsilon_{0.35f_{cc}}$$

(7) $$\varepsilon_{pl}=\varepsilon_{un}-\frac{\varepsilon_{un}+\varepsilon_{a}}{\sigma_{un}+E_{c}\varepsilon_{a}}\;\;where\;\varepsilon_{un}>\varepsilon_{0.35f_{cc}}$$

(8) $$\varepsilon_{a}=a\sqrt{\varepsilon_{un}\varepsilon_{cc}}$$

(9) $$a=\max\left\{\frac{\varepsilon_{cc}}{\varepsilon_{cc}+\varepsilon_{un}};\frac{0.09\varepsilon_{un}}{\varepsilon_{cc}}\right\}$$

(10) $$\sigma_{new}=0.92\sigma_{un}+0.08\sigma_{r0}$$

(11) $$\varepsilon_{re}=\varepsilon_{un}+\frac{\left(\sigma_{un}-\sigma_{new})(2+\frac{f_{cc}}{f_{c}}\right)}{E_{r0}}\;$$

(12) $$E_{r0}=\frac{\sigma_{r0}-\sigma_{new}}{\varepsilon_{r0}-\varepsilon_{un}}$$

The plastic strain was obtained from the regression analysis of the test. According to the test data, the plastic strain was only related to the unloading point and had little relationship with the lateral compressive stress level (further research should be undertaken).

The equation of unloading stress–strain curve of confined concrete was

(13) $$\sigma_{c}=\sigma_{un}-\frac{r_{un}x_{un}}{r_{un}-1+x_{un}^{r_{un}}}\sigma_{un}$$

(14) $$x_{un}=\frac{\varepsilon_{c}-\varepsilon_{un}}{\varepsilon_{pl}-\varepsilon_{un}}$$

(15) $$r_{un}=\frac{2k_{r}E_{c}}{2E_{c}-E_{sec\_un}}$$

(16) $$E_{sec\_un}=\frac{\sigma_{un}}{\varepsilon_{un}-\varepsilon_{pl}}$$

The formulations of the above unloading curve were similar to that of the cement concrete reinforced with stirrups or steel tubes [35]. It can be explained that confined constant stress resulted from yielded steel materials was equal to the confined stress applied in the triaxial test. The reloading branch of the cyclic stress–strain curve consisted of nonlinear and linear branches:

(17) $$1\mathrm{st}\;\mathrm{branch}:\;\sigma_{c}=\sigma_{r0}+\sqrt{2}(\sigma_{new}-\sigma_{r0})\ln(\frac{\varepsilon -\varepsilon_{r0}}{\varepsilon_{un}-\varepsilon_{r0}}+1)$$

(18) $$2\mathrm{nd}\;\mathrm{branch}:\;\sigma_{c}=\sigma_{re}-\frac{\sigma_{re}-\sigma_{new}}{\varepsilon_{re}-\varepsilon_{un}}(\varepsilon_{re}-\varepsilon )$$

It was noted that the nonlinear curve model was used in both of the first portion and transition portion [18], but based on the experimental data, the transition portion in this study was simplified and modified to a linear model.

**Figure 10:**
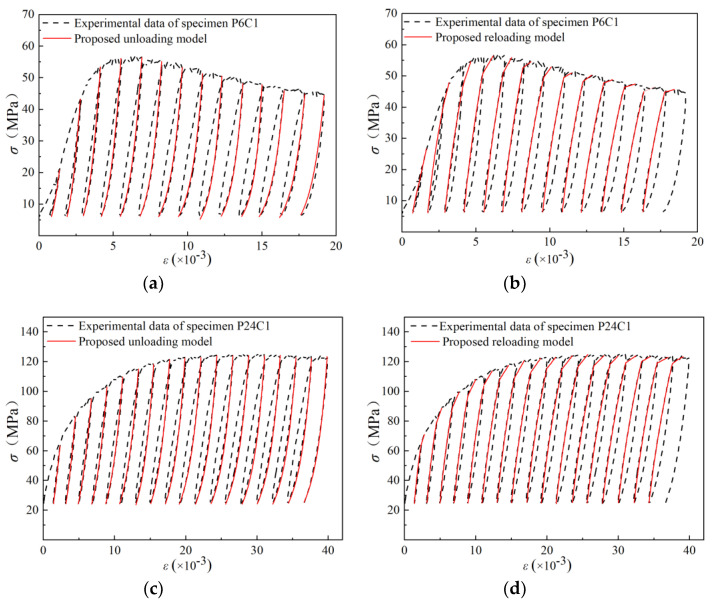
Cyclic axial stress–strain relation predictions of confined GPC: (**a**) unloading curves prediction of P6C1; (**b**) reloading curves prediction of P6C1; (**c**) unloading curves prediction of P24C1; (**d**) reloading curves prediction of P24C1.

The comparison between the experimental results of P6C1 and P24C1 with the axial cyclic stress–strain model above was shown in Figure 10. It can be seen that the stress–strain behavior predicted by the model was close to the test results under different confining pressures. The residual plastic strains predicted by the unloading curve model was consistent with the test results, and the predictions of transition point and reloading strain also matched with the test points closely on the reloading curve. Considering the change of GPC properties, further experimental studies should be carried out to improve the proposed model, such as establishing the relationship between residual plastic strain, reloading strain, and uniaxial compressive strength grade of GPC [40,41,42].

### 3.5. The Energy Absorption and Specific Toughness under Uniaxial and Triaxial Compression

Generally, the amplitude of energy per unit volume obtained from area under the stress–strain curve whose strain value can reach a certain value (often failure strain) is called energy absorption. However, the larger the confined stress level is, the more difficult it is to obtain the complete stress–strain curve. For example, when the confining stress is up to 18 MPa, due to the limitation of measuring equipment, only the rising section of the stress–strain curve can be obtained and an obvious descending trend is not witnessed. In this study, energy absorption capacity (*W*) is the area under the curve ranging from the beginning of the stress–strain curve to 85% of peak stress in the descending section of the curve. Specific toughness *δ* is defined as the ratio of energy absorption capacity to peak stress of the same specimen. As is shown in the Table 5, the experimental *W* and *δ* are calculated by the method above when the confining stress was under 18 MPa, and all the theoretical *W* and *δ* are listed by the calculation of Mander’s model [34]. It can be concluded that the load path has little effect on the *W* and *δ*, both monotonic and cyclic loading result in the similar outcomes for the calculated confined stress level (6 MPa and 12 MPa). On the other hand, with the confining stress increasing, the *W* and *δ* substantially improved compared to the uniaxial compression without confining stress. Under the monotonic loading condition, the confining stress varied from 6 to 12 MPa, the mean value of *δ*_exp_ was improved by 483% and 1106%, respectively, compared to zero confining stress. For larger confined stress levels (18 MPa and 24 MPa), the theoretical model provided a prediction that 2057% and 2356% growth rate can be, respectively, inferred compared to the theoretical value of zero confining stress monotonic loading.

## 4. Conclusions

In this study, actively confined GPC cylindrical specimens were compressed under monotonic and cyclic loading. The following conclusions can be drawn according to the experimental results and discussion:

1. The loading path had little effect on the axial peak strength and strain of confined GPC. In the triaxial test, the GPC with brittleness turns into a ductile material when the confined stress ratio (*σ*_l_/*f*_c_) exceeded 0.4. The peak stress prediction model proposed by Lim et al. [31] for cement concretes can also accurately predict the peak strength of confined GPC, which showed that GPC and cement concrete had a similar strength enhancement coefficient. However, the strain enhancement coefficient of GPC was lower than that of cement concrete under a similar confinement level.
2. The constitutive behavior of actively confined GPC was similar to that of the conventional cement concrete. Under the condition of same confinement level, the envelope curves under cyclic compression were consistent with the stress–strain curves under monotonic loading, which can be simulated by Mander’s model with enough accuracy. This means the model provided by Mander et al. for conventional cement concrete was proved to be a necessary material model to study the mechanical behavior of actively confined GPC.
3. A cyclic axial compressive stress–strain model composed of a monotonic envelope curve (modified Mander’s model) and cyclic hysteresis curve (modified Lokuge’s model) can be used for the constitutive behavior of actively confined GPC under cyclic loading. The comparison among the experimental results showed that the model accurately predicted the residual plastic strain, the transition point, and reloading strain of GPC with active confinement, and fully simulated the degradation of stiffness caused by unloading/reloading cycles, which was consistent with the experimental results.
4. The energy absorption and specific toughness were calculated and discussed by both monotonic and cyclic stress–strain curves. The presented values and the discussion of the results could provide important indications for future studies.

## Figures and Tables

**Table 1:** Properties and compositions of the fly ash (FA) and granulated blast furnace slag powder (GBFS) (wt.%).

Binding Materials	SiO_2_	Al_2_O_3_	CaO	Fe_2_O_3_	TiO_2_	K_2_O	SO_3_	MgO	Na_2_O	L.O.I
FA	49.10	36.70	4.96	3.67	1.39	0.943	0.49	0.37	0.20	2.08
GBFS	32.28	13.80	37.85	2.74	1.30	0.96	2.90	3.73	0.70	1.30

**Table 2:** Mixture proportions of geopolymer concrete (GPC) used in this study (kg/m^3^).

FA	GBFS	SS	SH	Sand	CA	Added W	SP
234	156	122	49	764	1023	11.5	5.4

**Table 3:** Measured peak values of axial stress and strain of the tested specimens.

Load Path	Confining Lateral Stress *σ*_l_ (MPa)	Peak AXIAL Stress *f*_cc_ (MPa)	Peak Axial Strain *ε*_cc_ (×10^−3^)
Uniaxial	0	27.13	2.18
Uniaxial	0	28.86	2.23
Uniaxial	0	31.49	2.38
P6M1	6	57.01	7.02
P6M2	6	57.04	7.13
P6C1	6	54.70	5.73
P6C2	6	59.40	7.63
P12M1	12	81.14	12.01
P12M2	12	78.66	11.11
P12C1	12	79.23	11.68
P12C2	12	83.46	13.04
P18M1	18	106.02	20.95
P18M2	18	102.50	19.60
P18C1	18	100.89	18.23
P18C2	18	101.94	18.28
P24M1	24	128.38	26.60
P24M2	24	124.27	25.51
P24C1	24	122.88	23.51
P24C2	24	125.91	28.52

The samples in Table 3 were identified with the letter P followed by a digit indicating the confining pressure value, and the letter M or C denoted monotonic or cyclic compression, respectively. In each sample design, two to three identical samples were tested and marked with 1 or 2 after the last letter.

**Table 4:** Prediction models of peak axial stress and strain.

Concrete Type	Source	Analytical Expressions of *f*_cc_ and *ε*_cc_
High-strength cement concrete	Ansari et al. [28]	$\begin{array}{l} f_{cc}=f_{c}[1+2.45(\frac{\sigma_{l}}{f_{c}}{)}^{0.703}]\;\;\\ \varepsilon_{cc}=\varepsilon_{0}(1+15.15\frac{\sigma_{l}}{f_{c}})\; \end{array}$
High-strength cement concrete	Candappa et al. [29]	$\begin{array}{l} f_{cc}=f_{c}(1+5.3\frac{\sigma_{l}}{f_{c}})\;\;\\ \varepsilon_{cc}=\varepsilon_{0}(1+20\frac{\sigma_{l}}{f_{c}})\; \end{array}$
High-strength cement concrete	Xiao et al. [30]	$\begin{array}{l} f_{cc}=f_{c}[1+3.24(\frac{\sigma_{l}}{f_{c}}{)}^{0.8}]\;\;\\ \varepsilon_{cc}=\varepsilon_{0}[1+17.4(\frac{\sigma_{l}}{f_{c}}{)}^{1.06}]\; \end{array}$
Normal-and light-weight cement concretes	Lim et al. [31]	$\begin{array}{l} f_{cc}=f_{c}+5.2f_{c}^{0.91}(\frac{\sigma_{l}}{f_{c}}{)}^{a}\;\\ where\;a=f_{c}^{-0.06}\;\;\\ \varepsilon_{cc}=\varepsilon_{0}+0.045(\frac{\sigma_{l}}{f_{c}}{)}^{1.15}\; \end{array}$
Geopolymer concretes	Haider et al. [32]	$\begin{array}{l} f_{cc}=f_{c}[1+3.3(\frac{\sigma_{l}}{f_{c}}{)}^{0.8}]\;\;\\ \varepsilon_{cc}=\varepsilon_{0}[1+11.6(\frac{\sigma_{l}}{f_{c}}{)}^{1.06}]\; \end{array}$
Ambient- and oven-cured geopolymer concretes	Aliakbar et al. [17]	$\begin{array}{l} f_{cc}=f_{c}+5.2f_{c}^{0.91}(\frac{\sigma_{l}}{f_{c}}{)}^{a}\\ where\;a=f_{c}^{-0.06}\;\;\\ \varepsilon_{cc}=\varepsilon_{0}+0.045(\frac{\sigma_{l}}{f_{c}}{)}^{1.15}\; \end{array}$

*f*_cc_ and *ε_cc_*, respectively, denote peak stress and peak strain with lateral confinement; *f*_c_ and *ε*_0_, respectively, denote uniaxial compressive strength and uniaxial peak strain; *σ*_l_ denotes lateral confined stress.

**Table 5:** Measured energy absorption ability and specific toughness of the tested specimens.

Specimen Type	*W*_exp_(10^−3^)	*W*_the_(10^−3^)	*δ*_exp_(10^−3^)	*δ*_the_(10^−3^)
P0M1	72.70	80.57	2.50	2.77
P0M2	70.15		2.41	
P0M3	73.20		2.52	
P6M1	671.62	740.85	11.78	12.99
P6M2	695.35		12.20	
P6C1	680.42		12.44	
P6C2	672.25		11.32	
P12M1	2242.64	2232.15	27.82	27.69
P12M2	2180.54		27.05	
P12C1	2250.24		28.40	
P12C2	2180.56		26.13	
P18M1	N/A	5904.01	N/A	57.411
P18M2	N/A		N/A	
P18C1	N/A		N/A	
P18C2	N/A		N/A	
P24M1	N/A	8182.56	N/A	65.27
P24M2	N/A		N/A	
P24C1	N/A		N/A	
P24C2	N/A		N/A	

The *W*_exp_ and *δ*_exp_, respectively, denote the energy absorption ability and specific toughness of experimental data; the *W*_the_ and *δ*_the_, respectively, denote the energy absorption ability and specific toughness of theoretical data.
